# Data of annotated draft genome assemblies of three bacterial isolates from the ‘Panchabrihi’ rice, soybean, and bean-associated niches in Bangladesh

**DOI:** 10.1016/j.dib.2026.113081

**Published:** 2026-07-16

**Authors:** Nusrat Sultana, Abed Chaudhury, Saria Farheen

**Affiliations:** a Department of Botany, Faculty of Life and Earth Sciences, Jagannath University, Dhaka, 1100, Bangladesh; b Krishan Foundation (ht tps://krishanfoundation.com), Hazipur Village, Moulvibazar 3200, Sylhet, Bangladesh; c Department of Microbiology, BRAC University, Dhaka, 1212, Bangladesh

**Keywords:** Abed Chaudhury, Whole-genome sequencing, PRJNA1366070

## Abstract

We have collected the bacterial communities inhabiting the rhizosphere and seed-surface of selected crop plants (‘Panchabrihi’ rice, soybean, and bean) in Bangladesh and created a draft genome assembly database of the isolated bacterial strains. Three bacterial isolates were : A1 from the rhizosphere of Panchabrihi rice, A7 and A10 from the surface of soybean and bean seeds. Whole-genome sequencing, draft genome assembly, BLAST comparison with reference genomes, Nucleotide Identity (ANI), digital DNA-DNA hybridization (dDDH), Average Amino Acid Identity (AAI), phylogenomic analyses, and annotations using Bakta revealed that the isolates are *Bacillus cereus* A1, *Bacillus subtilis* A7, and *Acinetobacter soli* A10. Paired-end Illumina sequencing data, draft genome assembly, and annotations generated were deposited in the NCBI database under BioProject PRJNA1366070. Afterwards, we have analysed the potential of plant growth–promoting (PGP) capacities using the eggNOG-mapper analysis, COG functional classification, secondary metabolite biosynthetic gene cluster analysis using antiSMASH, and KEGG Orthology (KO) completeness analysis. After manual curation of the functional properties of the predicted genes, we found that all three genomes were associated with PGP functions, including nutrient solubilization, phytohormone production, stress tolerance, and root colonization. Additionally, we have also analysed the antibiotic resistance genes (ARGs), mobile genetic elements, virulence-associated genes, toxin genes, and pathogenicity genes of the isolated strains, and generated the respective database. The draft genomes were also found to have several antibiotic resistance determinant genes, mobile genetic elements, virulence-associated genes, and human pathogenic genes. Therefore, we have performed a comparative biosafety risk assessment on all three genomes and concluded that *Bacillus subtilis* A7 (rather than the A1 and A10) will be a safer option for possible sustainable agricultural practices. We have generated a comprehensive metadata table and submitted all relevant databases to Zenodo. Our databases will be useful for future identification of the potential PGP bacterial strains and for assessing the biosafety traits before agricultural application.

Specifications Table

**Table:** 

Subject	Earth & Environmental Sciences
Specific subject area	Rhizosphere and seed-surface-associated bacterial genomics
Type of data	Table, Image, Chart, Graph, Figure, Raw, Analysed, Filtered, Processed, details protocol
Data collection	Rhizosphere and seed-surface-associated bacteria were collected from rice, bean, and soybean plants in the Sylhet region of Bangladesh. Pure cultures of the bacterial strains were obtained through various plating methods, and their DNA was extracted using the SDS-phenol-chloroform method. Whole-genome sequencing was conducted on an Illumina NextSeq 2000 platform using a 300-cycle paired-end protocol (BDGenomes, Savar, Dhaka). The draft genome assembly, comparison with the reference genome, and annotation were performed using various bioinformatics software, and the related data were deposited in the NCBI database under BioProject PRJNA1366070. In addition to that, the potential plant growth-promoting trait, antibiotic resistance genes (ARGs), mobile genetic elements, virulence-associated genes, toxin genes, and pathogenicity-related genes of the isolated strains were also analysed, and the associated data were deposited in Zenodo with DOI 10.5281/zenodo.20935544 (https://zenodo.org/records/20935544).
Data source location	The bacterial isolates were collected from the Sylhet region of Bangladesh, with the GPS coordinates of 24° 53′ 41.2872′' N and 91° 52′ 8.5224′' E. The associated sequencing data, draft genome assembly, and annotations are deposited in NCBI and other databases can be found on Zenodo DOI 10.5281/zenodo.20935544 (https://zenodo.org/records/20935544).
Data accessibility	BioSamples, the paired-end Illumina raw sequencing reads (NCBI Sequence Read Archive (SRA), WGS, draft genome assemblies, and annotations have been deposited in the NCBI database under BioProject accession number PRJNA1366070 (https://www.ncbi.nlm.nih.gov/bioproject/?term=PRJNA1366070) and File 1–4 are publicly available in Zenodo with DOI 10.5281/zenodo.20935544 (https://zenodo.org/records/20935544).
Related research article	None

## 1 Value of the Data

• We have screened the rhizosphere of ‘Panchabrihi’ (genotype K06) rice, and seed-surface of bean, and soybean for plant growth-promoting bacteria. Pachabrihi rice is actually considered an extraordinary invention of rice cultivation method, where five varieties of four-season rice can be cultivated from a single rice tree. In this study, therefore, we emphasized that these databases constitute an important resource for future search of plant growth-promoting traits, comparative genomics, and the identification of candidate genes potentially involved in plant–microbe interactions.
• Nowadays, plant growth-promoting bacteria are considered an eco-friendly alternative for sustainable agriculture instead of using chemical fertilizers and fungicides. However, formulating such an alternative is still a challenge. We therefore aim to screen the natural diversity of microbial communities of Bangladesh to enrich our knowledge of PGP bacteria.
• Our genome data has significant potential for continued research and technological advancements, not only on PGP bacterial communities that are essential as the field steadily progresses, but also the interaction between bacterial communities and ecological niches.

## 2 Background

Plant growth–promoting rhizobacteria (PGPR) are beneficial root-associated bacteria that enhance plant growth by facilitating nutrient uptake, synthesizing phytohormones, and suppressing soil-borne pathogens. Among the most widely studied PGPR species, those belonging to the genera *Bacillus* and *Pseudomonas* are particularly notable for their strong plant growth–promoting activities [1,2]. Several PGPR-based inoculants have now become commercially available to support sustainable agricultural practices; however, optimizing their formulation, stability, and field performance remains a significant challenge [3].

Bacterial isolates were obtained from the rhizosphere of rice (‘Panchabrihi’ genotype K06), soybean, and bean plants cultivated in Bangladesh. These isolates were previously shown to exhibit multiple plant growth–promoting (PGP) traits [[1], [2], [3]]. To characterize their taxonomic identity and to investigate the genetic basis of their plant growth–promoting potential, whole-genome sequencing was performed on three representative isolates (Table 1).

**Table 1:** Genome data of three bacterial genomes, A1, A7, and A10 (annotation using the NCBI prokaryotic genome annotation pipeline (PGAP) [9]).

Genome information	*Bacillus cereus* A1	*Bacillus subtilis* A7	*Acinetobacter soli* A10
BioSample accession number	SAMN53301951	SAMN53301952	SAMN53301953
Isolation source	Rhizosphere of Panchabrihi rice	Seeds of soybean	Seeds of bean
strain designation	A1	A7	A10
GPS coordinates	24° 53′ 41.2872″ N 91° 52′ 8.5224″ E	24° 53′ 41.2872″ N 91° 52′ 8.5224″ E	24° 53′ 41.2872″ N 91° 52′ 8.5224″ E
culture conditions	Luria–Bertani agar and incubated overnight at 37 °C	Luria–Bertani agar and incubated overnight at 37 °C	Luria–Bertani agar and incubated overnight at 37 °C
sequencing platform	Illumina NextSeq 2000	Illumina NextSeq 2000	Illumina NextSeq 2000
NCBI Sequence Read Archive (SRA) accession number	SRR36095922	SRR36095921	SRR36095920
Draft genome assemblies accession numbers	GCA_053894975.1	GCA_053895045.1	GCA_053894985.1
WGS accessions	JBSVMN000000000	JBSVMM000000000	JBSVML000000000
Taxonomic placement of assembled genome	cellular organisms; Bacteria; Bacillati; Bacillota; Bacilli; Bacillales; Bacillaceae; Bacillus; *Bacillus cereus* group	cellular organisms; Bacteria; Bacillati; Bacillota; Bacilli; Bacillales; Bacillaceae; Bacillus; *Bacillus subtilis* group	cellular organisms; Bacteria; Pseudomonadati; Pseudomonadota; Gammaproteobacteria; Moraxellales; Moraxellaceae; *Acinetobacter*)
Number of raw read pairs	954,844	2499,237	1821,575
Sequencing Depth/Coverage	45x	176x	154x
CheckM contamination percentage	1.18	15.29	56.8
CheckM completeness percentage	100	100	100
GC content	35	44	43
Number of reported contigs	215	85	712
Draft genome size (Mb) (without rounding)	5.3	4.6	3.7
Contig N50 (kb) (without rounding)	43.9	309	83.5
L50	33	6	15
Genes (total)	5656	4843	4312
CDSs (total)	5612	4782	4216
CDSs (with protein)	5256	4560	3793
hypothetical proteins	356	222	423
Genes (RNA)	44	61	96
rRNAs	6, 2, 2 (5S, 16S, 23S)	2, 5, 1 (5S, 16S, 23S)	8, 6, 6 (5S, 16S, 23S)
tRNAs	29	48	71
ncRNAs	5	5	5
Pseudo Genes (total)	356	222	423
CRISPR Arrays	0	0	3
percentages of complete single-copy BUSCOs	433	399	694
duplicated BUSCOs	7	41	78
fragmented BUSCOs	8	8	7
missing BUSCOs	2	2	3
mobile genetic elements (Total)	111	150	134
PlasmidFinder report	None	None	None
Probability of being a human pathogen	0.827	0.239	0.761
CARD Genes (Total)	14	25	3
virulence-associated genes	Virulence Genes for *Listeria* (clpB, FlaA, relA, tcsA)	Virulence Genes for *Listeria* (clpc, recA)	None
toxin genes	None	None	None
Identified secondary metabolite regions	Bacillibactin, Nostocyclopeptides, Petrobactin, Molybdenum Cofactor (Moco)	Surfactin, Plipastatin, Bacilysin, Bacillibactin, Subtilosin, Subtilisin, Fengycin, Bacillaene	Plipastatin, Acinetoferrin, Berninamycin, Bacillomycin
Xenobiotic degradation	No	No	Yes
Biosafety risk level	High caution	Medium or low	High (but risky)

Our study aimed to generate a publicly available draft genome assembly and the annotations for the potential plant growth-promoting bacterial strains from Bangladesh collected from some economically important crop plants, such as rice, bean, and soybean. The publicly available genomic data from this study will directly benefit PGPR-based agricultural practices associated with Panchabrihi rice and other crop-associated environments and future research efforts on the PGPR bacterial genome.

## 3 Data Description

BioSamples, the paired-end Illumina raw sequencing reads (NCBI Sequence Read Archive (SRA), WGS, draft genome assemblies, and annotations have been deposited in the NCBI database under BioProject accession number PRJNA1366070 (https://www.ncbi.nlm.nih.gov/bioproject/?term=PRJNA1366070) and other related data are publicly available in Zenodo with DOI 10.5281/zenodo.20935544 (https://zenodo.org/records/20935544). and these data are arranged as follows: Isolation technique of bacterial communities in File 1, Pre-decontamination data in File 2, Post-decontamination _data in File 3, and Comparative metadata in File 4.

## 4 Experimental Design, Materials and Methods

Bacterial colonies from rice roots were isolated by rinsing 1–2 g of root fragments in sterile water, followed by suspension in 9 mL phosphate-buffered saline. Samples were vortexed for 2–5 min and shaken at 150 rpm for 10 min. Serial 10-fold dilutions were prepared and spread plated on Luria–Bertani agar. In contrast, bacterial inocula from fresh soybean and bean seeds were aseptically isolated from seed surfaces and incubated overnight at 37 °C on nutrient agar plates (File 1). The isolated pure bacterial colonies are being preserved in our institutional culture collection, ensuring their long-term preservation.

Genomic DNA was extracted using the adapted standard SDS-phenol–chloroform method [4]. Cell lysis was achieved with a lysis buffer containing sodium dodecyl sulfate (SDS), followed by purification with phenol–chloroform–isoamyl alcohol to remove cellular debris. DNA was subsequently precipitated using ethanol and salt, yielding high-quality genomic DNA for sequencing (File 1).

Whole-genome sequencing was conducted on an Illumina NextSeq 2000 platform using a 300-cycle paired-end protocol (BDGenomes, Savar, Dhaka). The sequencing data were deposited in the NCBI database under BioProject accession number PRJNA1366070. A total of 954,844, 2499,237, and 1821,575 paired-end reads were generated for isolates A1, A7, and A10, respectively. Raw sequence quality was assessed using FastQC (Version 0.12.1), and Fastp (Version 1.0.1) [5] was applied for global trimming to correct sequence biases and to filter out low-quality reads (File 2.1). High-quality reads were assembled de novo using MEGAHIT (Version 1.2.9) [6], retaining contigs ≥1500 bp (File 2.2). Important to mention here that MEGAHIT was selected for this study because it provides efficient de novo assembly of short-read sequencing data, with acceptable completeness and contamination metrics useful for downstream genome analyses.

Assembly quality was evaluated using QUAST Version v5.0.2) [7] and BUSCO (Version v5.2.2) [8] with BUSCO v5 lineage datasets. Default parameters were used for all software unless otherwise specified. Essential metadata information on genome assemblies is provided in File 4. QUAST analysis, performed using the closest available reference genomes, showed that 81% of the A1 assembly aligned to the *Bacillus cereus* ATCC 14,579 Strain P1 reference genome, while 93% and 84% of the A7 and A10 assemblies aligned to *Bacillus subtilis XF-1* and *Acinetobacter soli* Strain M3–1–68, respectively, based on the genome fraction (%) metric. BLAST (BLAST+ 2.16.0) comparisons confirmed assembly gaps, which were more pronounced in A7 and A10 than in A1 (File 2.3). BUSCO completeness scores were 97% for A1 and 88% for A7, with only 1.8% genes fragmented. BUSCO analysis initially failed for A10 due to contamination.

Therefore, we have performed the contamination check on all the assembled genomes using CheckM (Version 1.2.5+galaxy0) (File 2.5) [9]. We found that 1.18% of the A1, 15.29% of the A7, and 56.85% of the A10 assembled genomes were initially contaminated. Since no direct experimental evidence is available to distinguish the contamination source, and our studied genomes showed 100% completeness, even though with some contamination, we avoided further investigation (File 1, Table 1). Meanwhile, we have removed contamination using NCBI FCS-GX (Version 0.5.5) [10] and MetaBAT2 (Version 2.18.23+galaxy0) [11], and all the contamination-free contigs were submitted in NCBI database (File 2.4 and 2.6).

We have generated comprehensive genome assembly statistics citing important metrics such as total assembly size, GC content, number of contigs, N50, L50, maximum contig length, sequencing depth/coverage, contamination estimates, BUSCO values and completeness percentages for all genomes calculated using QUAST (Version v5.2.0) and BUSCO (Version v5.2.2) (File 4, Table 1). Initial species identification of the isolates was performed using QUAST and BLAST-based sequence similarity searches in Proksee (https://proksee.ca/) [12]. These were further validated using a genome-wide comparative analysis method, such as Average Nucleotide Identity (ANI) calculation using FastANI (Version v1.33) [13] against closely related reference genomes, digital DNA–DNA hybridization (dDDH) values were obtained using the Type (Strain) Genome Server (TYGS), which applies the Genome BLAST Distance Phylogeny (GBDP) approach and strain-level identification using phylogenomic relationships using TYGS (File 3 and File 4) [14]. Genome annotations were performed using the NCBI Prokaryotic Genome Annotation Pipeline (PGAP, version 6.8) [15] and Bakta (Version v5.1) [16]. We identified 5612, 4782, and 4216 coding sequences in A1, A7, and A10, respectively. (File 3.1–1.6).

Functional annotation of protein-coding sequences extracted using Bakta (Version v5.1) were performed using eggNOG-mapper (Version v2.1.12) [17], from the assembled genomes of isolates A1, A7, and A10. KEGG Orthology (KO) lists derived from eggNOG-mapper outputs were used for KEGG pathway completeness analysis [18]. Only the KEGG pathway that were 50% complete were used for further manual curation in various functional genes potentially involved in plant growth-promoting (PGP) mechanisms, including nutrient cycling, phytohormone biosynthesis, and stress response pathways. Moreover, secondary metabolite biosynthetic gene cluster analysis using antiSMASH [19] were performed, revealed several secondary metabolite-specific gene clusters. Our results show the genomic evidence of plant-associated beneficial traits in all three isolates. However, we acknowledge that all the prediction exclusively derived from a bioinformatics pipeline rather than experimental validation. Therefore, we have extended our analysis of plant growth–promoting traits using PLaBA-db [20] in the reference genomes. The results showed that 31–39% of genes of the respective reference genomes were associated with PGP functions, predominantly related to root colonization, followed by stress tolerance, biofertilization, bioremediation, and plant immune responses (File 3.7–3.12)

To evaluate the biosafety profile of the bacterial isolates A1, A7, and A10, a comprehensive in silico analysis was performed focusing on antibiotic resistance genes (ARGs), mobile genetic elements, plasmid localization, and virulence-associated traits. Genome assemblies were analyzed using Proksee (https://proksee.ca/) for visualization and genomic context interpretation of ARGs, mobile genetic elements, and plasmid-associated regions, to visualize the potential chromosomal or putative plasmid localization of resistance determinants. In addition, pathogenicity [21] and virulence-related predictions [22] were performed using tools available through the Center for Genomic Epidemiology (CGE), Technical University of Denmark web server (https://cge.food.dtu.dk/services/). Antibiotic resistance gene prediction using the CARD database using Proksee (Version 1.2.0) [12] revealed multiple resistance determinants, including vancomycin resistance genes. Although no plasmid-associated region was found, several mobile genetic elements were detected in proximity of the antibiotic resistance genes. Moreover, although there were no genes related to toxins in all the analysed bacterial genomes, *Bacillus cereus* A1 and *Acinetobacter soli* A10 were found to have several pathogenic genes related to humans (File 3.13–3.16).

Overall, the combined bioinformatic framework provides a preliminary genome-based biosafety assessment, where Bacillus subtilis A7, could be a better option for further investigation for possible agricultural application compared to the other two strains. However, all findings are predictive in nature and should be interpreted with caution, as experimental validation is required to confirm functional activity and actual pathogenic potential (File 3.17).

## Limitations

We acknowledge several limitations of this study, such as the absence of long-read sequencing, fragmented contigs, the lack of experimental validation of PGP genes, and contamination issues. Therefore, future research will be focused on this area to complement this data article.

## Ethics Statement

The current work does not involve human subjects, animal experiments, or any data collected from social media platforms.

## CRediT Author Statement

**Nusrat Sultana**: Conceptualization, Methodology, Software. Data curation, Writing, Original draft preparation, editing, and revision; **Saria Farheen**: Methodology and Investigation; **Abed Chaudhury**: Supervision, funding acquisition.

## Data Availability

Original DataData of annotated draft genome assemblies of three bacterial isolates from the 'Panchabrihi' rice, soybean, and bean-associated niches in Bangladesh (Original data). Research DataGenome analysis of three bacterial isolates from the rhizosphere of Panchabrihi rice, soybean, and bean in Bangladesh (Original data).
